# Bevacizumab in ovarian cancer: Clinical data and predictive and prognostic biomarkers

**DOI:** 10.1002/ctm2.70591

**Published:** 2026-01-10

**Authors:** Maria Rosaria Lamia, Erica Perri, Gustavo Baldassarre, Sandro Pignata, Chiara D'Alessio, Davide Limongello, Francesca Basso‐Valentina

**Affiliations:** ^1^ Department of Clinical Medicine and Surgery University Federico II Naples Italy; ^2^ Department of Urology and Gynecology Istituto Nazionale Tumori IRCCS Fondazione G Pascale Naples Italy; ^3^ Department of Precision Medicine Università Degli Studi della Campania Napoli Italy; ^4^ Molecular Oncology Unit, Centro di Riferimento Oncologico di Aviano IRCCS, National Cancer Institute Aviano Italy; ^5^ Department of Obstetrics and Gynecology AOUI Verona University of Verona Verona Italy

**Keywords:** angiogenesis, bevacizumab, homologous recombination deficiency (HRD), ovarian cancer, PARP inhibitors, predictive biomarkers, prognostic biomarkers, VEGF

## Abstract

Angiogenesis, driven by the vascular endothelial growth factor (VEGF)/VEGFR signalling axis under hypoxic conditions, is one of the hallmarks of ovarian cancer (OC), contributing to tumour progression, metastatic dissemination and immune evasion. Hypoxia‐induced angiogenic signalling sustains tumour growth and shapes an immunosuppressive tumour microenvironment, while homologous recombination deficiency (HRD) has been associated with increased tumour hypoxia and pro‐angiogenic signalling. Conversely, VEGF pathway inhibition may exacerbate DNA damage and modulate immune cell trafficking, providing a strong biological rationale for synergy between anti‐angiogenic agents, PARP inhibitors (PARPi), and immune checkpoint inhibitors. Bevacizumab, a humanised monoclonal antibody targeting VEGF‐A, represents a pivotal therapeutic agent in OC management by inhibiting tumour angiogenesis and inducing transient vascular normalisation. Its clinical efficacy has been demonstrated as maintenance therapy in the first‐line setting, alone or in combination with PARPi for HRD‐positive disease, and in the recurrent setting both in platinum‐sensitive and platinum‐resistant disease. Despite these benefits, variability in patient response highlights the unmet need for validated predictive biomarkers. Circulating, tissue‐based and molecular biomarkers have been investigated, including angiogenic factors (Tie2/Ang1 axis, interleukin‐6 [IL‐6] and chitinase‐3‐like protein [YKL‐40]), VEGF‐A isoforms, microvessel density, EGFR/ADAM17 signalling, angiomiRs and transcriptional subtypes with mesenchymal and proliferative phenotypes showing greater sensitivity to anti‐angiogenic strategies. Although HRD status holds prognostic relevance and selected microRNAs show emerging potential, no biomarker has yet been validated to predict benefit from bevacizumab in clinical practice. Translational analyses from the MITO16A/MaNGO OV‐2 program, highlight challenges such as assay standardisation, multiplicity correction and external validation, while identifying tumour immune infiltration patterns, TP53 mutation classes and composite HRD assessments as areas of further investigation. In conclusion, bevacizumab remains an integral component of OC treatment. Future progress will depend on biomarker‐driven, prospectively designed clinical trials and the integration of multi‐omic data and machine learning approaches to enable precision application of anti‐angiogenic strategies, maximising clinical benefit while minimising toxicity.

## Graphical abstract

Evidence supporting bevacizumab in ovarian cancer and predictive biomarkers: highlighting opportunities to improve patient selection and advance personalised treatment strategies.

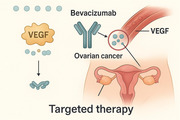

## 1 BACKGROUND

Worldwide, ovarian cancer (OC) ranks as the eighth most common cancer among women, accounting for an estimated 3.7% of all cancer cases and 4.7% of cancer‐related deaths in 2020.^1^ Approximately 25% of OC diagnoses are hereditary: germline mutations in the BRCA1 and BRCA2 genes represent the most well‐established risk factors,^2^ while a minor subgroup is attributable to other gene variants, including DNA mismatch repair genes (Lynch syndrome) and those involved in the DNA double‐strand break repair system, such as CHEK2, RAD51, BRIP1 and PALB2.^3^ The main recognised environmental risk factors are nulliparity or infertility, obesity, hormonal replacement therapy and endometriosis, which is strongly associated with clear cell and low‐grade endometrioid and serous OC histotypes.^4^ On the other hand, long‐term use of oral contraceptives (≥10 years) lowers risk by up to 60%^5^ and four full‐term pregnancies are associated with a roughly 40% risk decrease.^6^

**FIGURE 1:**
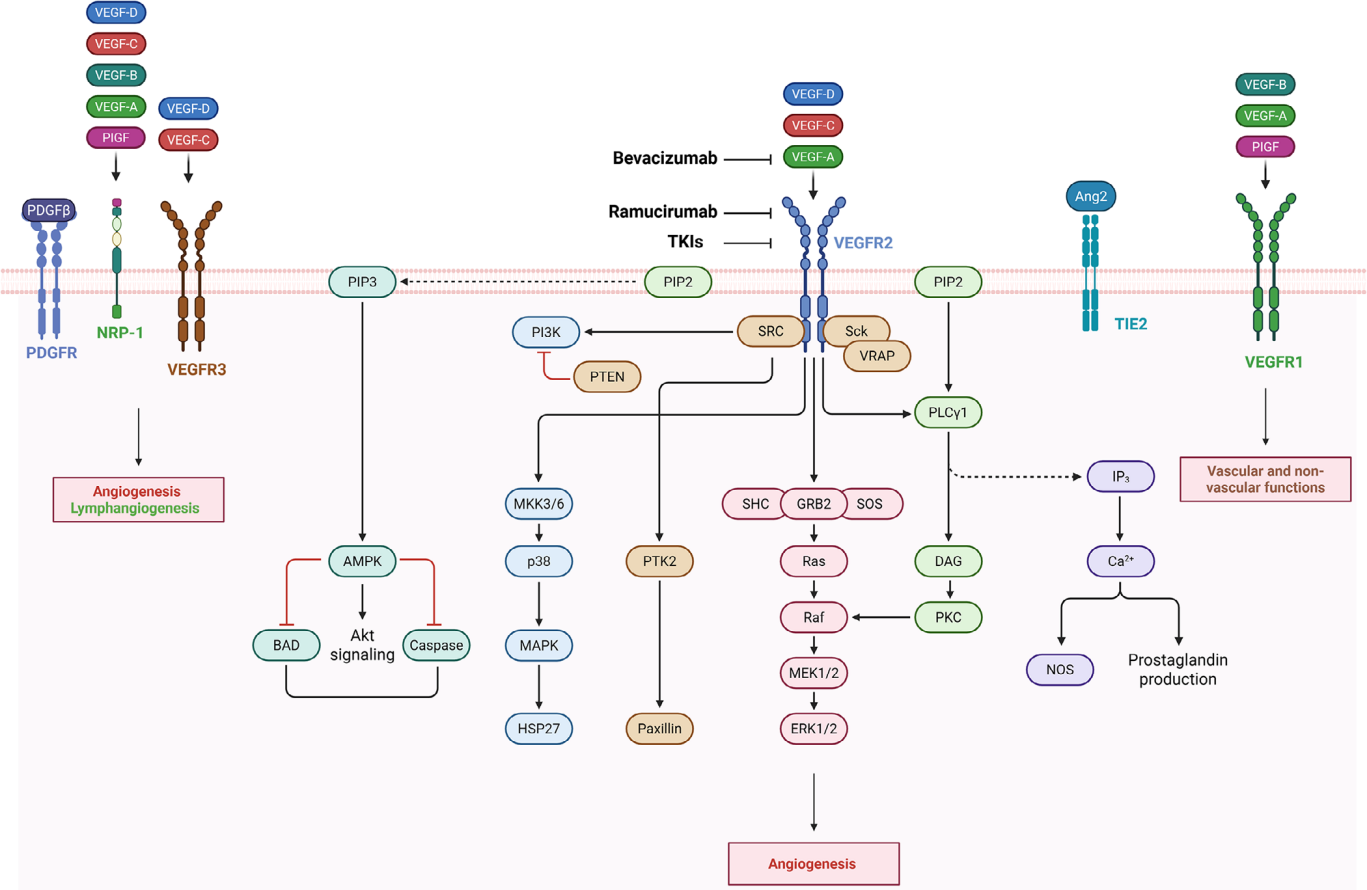
Molecular mechanisms of the vascular endothelial growth factor (VEGF)/VEGFR signalling axis and its intracellular pathways. Key ligands and receptors involved in angiogenesis, with a focus on pathways activated by the VEGF‐A/VEGFR‐2 axis and its inhibitors.

The pathogenesis of OC is still unclear; however, it is well established that angiogenesis is a critical driver of OC progression, supporting tumour growth and extensively remodelling the tumour microenvironment (TME). Central to this process is the vascular endothelial growth factor (VEGF)–VEGFR signalling axis, where VEGF binding to VEGFR‐2 activates the PI3K/AKT and RAS/MAPK pathways, promoting endothelial cell proliferation, migration, survival and neovascularisation^7^ (Figure 1). These angiogenic effects enhance tumour invasiveness by inducing epithelial‒mesenchymal transition (EMT), disrupting vascular integrity, and increasing permeability, factors that collectively contribute to ascites formation.^8^ VEGF also induces VE‐cadherin phosphorylation, weakening endothelial junctions and further aggravating vascular leakage.^9^

Hypoxic conditions within the TME exacerbate angiogenesis through the stabilisation of hypoxia‐inducible factor‐1α (HIF‐1α), which upregulates VEGF and perpetuates a feed‐forward loop between hypoxia and neovascularisation.^10^, ^11^ HIF‐1α also plays a key role in immune evasion by upregulating programmed death cell‐ligand 1 (PD‐L1), suppressing T‐cell activity and promoting the recruitment of regulatory T cells and tumour‐associated macrophages.^12^

Moreover, hypoxia‐induced glycolysis increases lactic acid production, acidifying the TME and impairing immune cell function. This acidic environment also activates matrix metalloproteinases, which degrade the vascular basement membrane and facilitate tumour invasion and metastasis.^13^ Emerging evidence also suggests that homologous recombination deficiency (HRD) promotes genomic instability and tumour hypoxia, resulting in pro‐angiogenic signalling, while concurrent VEGF pathway inhibition can further exacerbate DNA damage and potentiate PARP inhibitor (PARPi) sensitivity.^14^ Bevacizumab is a recombinant humanised IgG1 monoclonal antibody that specifically targets VEGF‐A isoforms, thereby inhibiting the VEGF‐mediated angiogenic signalling pathway.^15^

This blockade leads to inhibition of new capillary formation by suppressing endothelial proliferation, migration and tube formation; reduction of VEGF‐A‐mediated autocrine survival signalling within tumour cells; induction of ‘vascular normalisation’, characterised by tightening of endothelial junctions, increased pericyte coverage, decreased vessel permeability, improved perfusion, alleviation of hypoxia and enhanced delivery and efficacy of concomitant therapies.^16^

Nevertheless, there are some strategies that tumours develop to escape these therapies. Resistance to bevacizumab occurs through a spatiotemporal adaptation process where tumours bypass VEGF‐A blockade by activating redundant signalling pathways such as fibroblast growth factor (FGF), platelet‐derived growth factor (PDGF) and angiopoietins. To sustain growth, the TME recruits bone‐marrow‐derived cells and pericytes that stabilise alternative vascular networks. Furthermore, treatment‐induced hypoxia triggers aggressive mechanisms such as vessel co‐option by which tumour cells subvert pre‐existing healthy vasculature and the induction of EMT, which promotes invasion and metastasis.^17^

The aim of this review is to describe the data available on the clinical use of the antiangiogenic drug bevacizumab and of the biomarkers that have been investigated as possible prognostic or predictive factors in patients receiving these therapies.

## 2 BEVACIZUMAB IN FIRST‐LINE OVARIAN CANCER TREATMENT

In the evolution of OC therapy, bevacizumab remains a cornerstone. The GOG‐0218, published in 2011, was the first trial which demonstrated the benefit of adding bevacizumab to platinum‐based chemotherapy (PBCT). This double‐blind, placebo‐controlled study randomised 1873 patients with stage III (incompletely resectable) or stage IV OC following debulking surgery to one of three treatment arms: (1) chemotherapy plus placebo (control); (2) chemotherapy plus bevacizumab during cycles 2–6 followed by placebo (bevacizumab initiation); and (3) chemotherapy plus bevacizumab during cycles 2–22 (bevacizumab throughout). Compared to control, bevacizumab throughout significantly reduced the risk of progression or death (hazard ratio [HR]: .72; 95% confidence interval [CI]: .63–.82; *p* < .001), although overall survival (OS) did not significantly differ. Adverse events were more frequent in the bevacizumab groups, including hypertension and gastrointestinal‐wall disruption.^18^ These findings established bevacizumab as a key therapeutic agent in this setting.

The ICON7 trial, an open‐label phase III study, enrolled 1528 women with both early high‐risk and advanced‐stage newly diagnosed OC. Patients received standard PBCT with or without concurrent bevacizumab (7.5 mg/kg) followed by maintenance. Although no significant OS difference was observed in the overall population (restricted mean survival time [RMST] 45.5 vs. 44.6 months; *p* = .85), a significant benefit emerged in the poor‐prognosis subgroup (39.3 vs. 34.5 months; *p* = .03), represented by stage IV and stage III with residual disease at primary surgery.^19^

A groundbreaking trial integrating anti‐angiogenic therapy with a PARPi is the PAOLA‐1 study. This randomised, double‐blind phase III trial evaluated bevacizumab in combination with olaparib versus placebo as maintenance therapy in 806 patients who had responded to first‐line chemotherapy plus bevacizumab. The combination significantly improved median progression‐free survival (mPFS) compared to bevacizumab alone (22.1 vs. 16.6 months; HR: .59; *p* < .001), with particularly marked benefit in HRD‐positive patients (HR: .33 in BReast Cancer (BRCA)‐mutated tumours).^20^

These effects were observed independently from the residual tumour at primary surgery. Based on these findings, combining bevacizumab with olaparib is approved in patients with HRD‐positive tumours. The key limitation of the PAOLA‐1 trial is the control arm with bevacizumab and the lack of a comparison with PARPi alone. Consequently, it is not possible to clearly define the incremental benefit of adding bevacizumab to PARPi across the different HRD subgroups.

MITO25.1 is an ongoing trial, which is trying to overcome this limitation investigating the optimal first‐line treatment based on HRD status. HRD‐negative patients receive either bevacizumab or rucaparib maintenance; HRD‐positive patients receive rucaparib alone or with bevacizumab. Although no results are yet available, the trial highlights increasing interest in synergistic strategies involving anti‐angiogenic agents and PARPi.^21^

Several studies have explored the role of immunotherapy in combination with bevacizumab, based on the evidence that bevacizumab can increase lymphocytes trafficking in the tumour. The IMagyn050/GOG‐3015‐ENGOT‐ov39 trial tested atezolizumab added to chemotherapy and bevacizumab in 1301 newly diagnosed patients. No significant PFS or OS benefit was seen in the overall population, although a modest improvement was observed in PD‐L1‐positive patients (20.8 vs. 18.5 months; HR: .80; *p* = .038).^22^

The DUO‐O phase III trial further investigated triplet strategies in non‐BRCA‐mutated high‐grade OC. Patients were randomised in three arms to receive carboplatin + paclitaxel + bevacizumab (CP + B), CP + B with durvalumab followed by maintenance with durvalumab or CP + B + durvalumab followed by maintenance with durvalumab and olaparib. Arm 3 showed the most pronounced benefit of the combination of the bevacizumab and durvalumab in the HRD‐positive subgroup, with an mPFS of 45.1 months, the longest reported in first‐line therapy.^23^

Bevacizumab remains one of the standard options in first line OC in combination with chemotherapy or PARPi (Table 1). The potential synergy of bevacizumab with immune checkpoint inhibitors (ICIs) has not been demonstrated.

**TABLE 1:** Bevacizumab in first‐line ovarian cancer (OC) treatment.

Trial name	Patient population	Treatment arm	Control arm	Primary endpoint	Outcome
GOG‐0218^17^ (2011)	Stage FIGO III or IV OC after surgery (PDS or biopsy)	Bevacizumab initiation (PBCT + B cycles 2–6) Bevacizumab throughout (PBCT + B cycles 2–22)	PBCT + placebo	PFS	Bevacizumab throughout PFS (HR: .72; p < .001); no OS difference
ICON7^18^ (2011)	Early high‐risk and advanced‐stage disease after surgery (PDS or biopsy)	PBCT + B (7.5 mg/kg) followed by maintenance	PBCT alone	OS	No OS difference overall; improved OS in poor‐prognosis subgroup (39.3 vs. 34.5 months; p = .03)
PAOLA‐1^19^ (2019)	Stage FIGO III or IV OC with response to first‐line PBCT + B	Maintenance bevacizumab + olaparib	Maintenance bevacizumab + placebo	PFS	mPFS (22.1 vs. 16.6 months; HR: .59; p < .001)
MITO 25.1^20^ (ongoing)	Stage FIGO IIIB or IV OC	HRD‐negative: bevacizumab or rucaparib maintenance HRD‐positive: rucaparib ± bevacizumab	N/A	PFS	Ongoing
IMagyn050/GOG‐3015‐ENGOT‐ov39^21^ (2021)	Stage FIGO IIIB or IV OC	PBCT + B + atezolizumab	PBCT + B + placebo	PFS, OS	PFS in PD‐L1+ subgroup (20.8 vs. 18.5 months; HR: .80; *p *= .038)
DUO‐O^22^ (ongoing)	Non‐BRCA‐mutated OC	PBCT + B + durvalumab + olaparib maintenance	PBCT + bevacizumab (±durvalumab)	PFS	mPFS (45.1 months) in HRD‐positive subgroup with triplet combination

Abbreviations: HR, hazard ratio; HRD, homologous recombination deficiency; mPFS: median progression‐free survival; N/A, not assessed; OS, overall survival; PBCT, platinum‐based chemotherapy (carboplatin + paclitaxel); PBCT + B, carboplatin + paclitaxel + bevacizumab; PD‐L1, programmed death cell‐ligand 1; PDS, primary debulking surgery; PFS, progression‐free survival.

## 3 BEVACIZUMAB IN PLATINUM‐SENSITIVE AND PLATINUM‐RESISTANT RECURRENT OVARIAN CANCER TREATMENT

The efficacy of bevacizumab has been demonstrated both in platinum‐sensitive and platinum‐resistant recurrent OC settings. Its use is supported by numerous clinical studies, and several ongoing trials are currently investigating its role in combination with emerging therapeutic agents (Table 2).

**TABLE 2:** Bevacizumab in platinum‐sensitive and platinum‐resistant recurrent ovarian cancer (OC) treatment.

Trial name	Patient population	Treatment arm	Control arm	Primary endpoint	Outcome
OCEANS^23^ (2012)	Platinum‐sensitive recurrence, bevacizumab‐naïve	Carboplatin+ gemcitabine + bevacizumab followed by bevacizumab maintenance	Carboplatin + gemcitabine + placebo followed by placebo maintenance	PFS	mPFS: 12.4 vs. 8.4 months (HR: .48; *p *< .0001); ORR: 78.5% vs. 57.4%
AGO‐OVAR 2.21/ENGOT‐ov18^24^ (2020)	Platinum‐sensitive recurrence	Carboplatin + PLD + bevacizumab followed by bevacizumab maintenance	Carboplatin + gemcitabine + bevacizumab followed by bevacizumab maintenance	PFS	mPFS: 13.3 vs. 11.6 months (HR: .81; *p *= .012)
MITO16B/ENGOT‐OV17^25^ (2021)	Platinum‐sensitive recurrence, previously treated with bevacizumab	PBCT + bevacizumab continuation	PBCT	PFS	mPFS: 11.8 vs. 8.8 months (HR: .51; *p *< .001)
GLORIOSA^27^ (ongoing)	Platinum‐sensitive recurrence with high FRα expression	Maintenance: bevacizumab + mirvetuximab soravtansine	Maintenance: bevacizumab alone	PFS	Ongoing
ATALANTE/ENGOT‐ov29^28^ (2022)	Platinum‐sensitive recurrence	PBCT + bevacizumab + atezolizumab, followed by maintenance with atezolizumab + bevacizumab	PBCT + bevacizumab + placebo, followed by maintenance with placebo + bevacizumab	PFS	mPFS: 13.5 vs. 11.3 months (NS); PD‐L1+ subgroup: 15.2 vs. 13.1 months (NS)
AURELIA^29^ (2014)	Platinum‐resistant recurrence	Chemotherapy (PLD, paclitaxel or topotecan) + bevacizumab	Chemotherapy (PLD, paclitaxel or topotecan)	PFS	mPFS: 6.7 vs. 3.4 months (HR: .48; *p *< .001)

Abbreviations: CP + B: carboplatin + paclitaxel + bevacizumab; FRα, folate receptor α; HR, hazard ratio; mPFS: median progression‐free survival; ORR, objective response rate; PBCT, platinum‐based chemotherapy; PD‐L1, programmed death cell‐ligand 1; PFS, progression‐free survival; PLD, pegylated liposomal doxorubicin.

In platinum‐sensitive relapsed disease, defined as tumour recurrence occurring at least 6 months after the last dose of PBCT received, bevacizumab is indicated in combination with a platinum‐based regimen, specifically carboplatin plus gemcitabine. This recommendation is supported by the evidence shown in the OCEANS trial, which randomised patients to receive carboplatin plus gemcitabine in combination with either bevacizumab or placebo, which were continued as maintenance therapy until disease progression or unacceptable toxicity. This trial demonstrated a significant improvement in PFS with bevacizumab (12.4 vs. 8.4 months; HR: .48; *p *< .0001) and an increase in objective response rate (ORR) (78.5% vs. 57.4%) compared to chemotherapy alone. However, no statistically significant difference in OS was observed.^24^

The AGO‐OVAR 2.21/ENGOT‐ov18 trial was a multicentre, open‐label, phase III study that enrolled patients with platinum‐sensitive recurrent OC. Patients were randomised 1:1 to receive either carboplatin plus pegylated liposomal doxorubicin (PLD) in combination with bevacizumab, or carboplatin plus gemcitabine with bevacizumab. In both arms, bevacizumab was continued as maintenance therapy until disease progression or unacceptable toxicity. The trial demonstrated a significant improvement in PFS in the experimental arm: mPFS was 13.3 months with carboplatin–PLD–bevacizumab compared to 11.6 months with carboplatin–gemcitabine–bevacizumab (HR: .81; 95% CI: .68–.96; *p* = .012).^25^ This study established carboplatin–PLD–bevacizumab as a more effective regimen than the carboplatin–gemcitabine–bevacizumab combination in the platinum‐sensitive relapse setting.

The MITO16B (ENGOT‐OV17) phase III study investigated the efficacy of bevacizumab rechallenge in patients with platinum‐sensitive OC who had previously received bevacizumab in the first‐line setting and who have experienced a relapse at least 6 months after the last dose of PBCT. Participants were randomised to receive a PBCT regimen, with or without the continuation of bevacizumab until disease progression or unacceptable toxicity. Median PFS was 11.8 months in the bevacizumab arm compared to 8.8 months in the chemotherapy‐alone group (HR: .51; 95% CI: .41–.64; *p* < .001),^26^ demonstrating that re‐treatment with bevacizumab could be an optimal approach in this setting of disease.

More recently, following encouraging results from the phase Ib/II study, the PICCOLO trial, investigating the anti‐folate receptor α (FRα) conjugated antibody mirvetuximab soravtansine in platinum sensitive disease with ORR of approximately 51.9% and a mPFS of 6.9 months,^27^ the GLORIOSA trial is currently evaluating the efficacy of bevacizumab in combination with mirvetuximab soravtansine in patients with platinum‐sensitive recurrent OC, whose tumours exhibit high FRα expression (≥75% of viable tumour cells staining at 2+ intensity, confirmed by immunohistochemistry (IHC) using the Ventana FOLR1 CDx assay).^28^ After completion of induction with PBCT plus bevacizumab, patients are randomised to receive maintenance treatment with bevacizumab alone or the combination of bevacizumab and mirvetuximab soravtansine. The results of this study are awaited and have the potential to change clinical practice in the management of platinum‐sensitive recurrent OC.^29^

The ATALANTE (ENGOT‐ov29) trial was a phase III, randomised, double‐blind, placebo‐controlled study designed to determine whether the addition of the ICI atezolizumab to standard PBCT plus bevacizumab could improve outcomes in patients with platinum‐sensitive recurrent OC. Patients were randomised to receive atezolizumab or placebo along with bevacizumab and six cycles of physician‐chosen PBCT, followed by maintenance with atezolizumab and bevacizumab. In the overall study population, the combination achieved a mPFS of 13.5 months compared with 11.3 months in the control arm, a difference that did not reach statistical significance. Similarly, in the PD‐L1‐positive subgroup, mPFS was 15.2 versus 13.1 months, again without significant benefit.^30^

## 4 BEVACIZUMAB IN PLATINUM‐RESISTANT RECURRENT OVARIAN CANCER TREATMENT

In patients with platinum‐resistant relapse, defined as disease recurrence occurring within 6 months after the last cycle of PBCT received, the AURELIA trial evaluated the addition of bevacizumab to chemotherapy, either liposomal doxorubicin, weekly paclitaxel or topotecan, compared with chemotherapy alone in patients with advanced platinum‐resistant OC. This study has shown a significant improvement, with a mPFS of 6.7 months in patients receiving the combination compared to 3.4 months in the arm of chemotherapy alone (HR: .48; *p* < .001), with a manageable safety profile, although predictable adverse events.^31^

Taken together, the studies discussed highlight the pivotal role of bevacizumab in the treatment of OC across different clinical settings. A true advancement in the field would be the identification of predictive biomarkers, which could enable a more precise selection of patients most likely to benefit from bevacizumab therapy.

### 4.1 Future combination of bevacizumab in ovarian cancer

**FIGURE 2:**
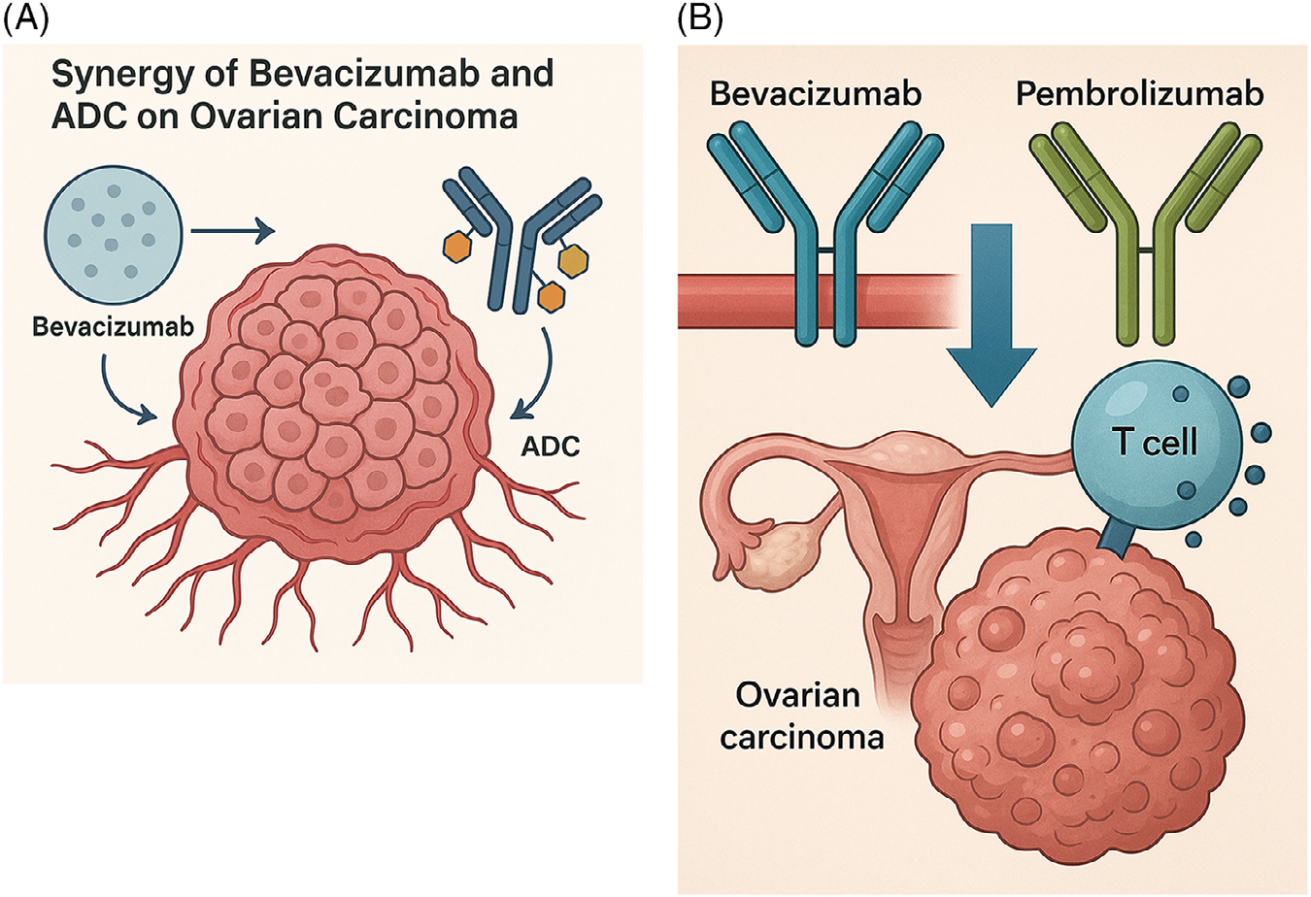
Synergistic mechanism of bevacizumab and antibody‒drug conjugates (ADCs) and pembrolizumab in ovarian cancer (OC). (a) This diagram illustrates how bevacizumab promotes vascular normalisation, enhancing the intratumoural delivery and penetration of ADCs. (b) This figure illustrates how bevacizumab mediates vascular normalisation and promotes T‐cell infiltration into the tumour microenvironment. This mechanism amplifies the activity of immune checkpoint inhibitors such as pembrolizumab, amplifying the immunosuppressive microenvironment of OC.

The combination of bevacizumab with new generation drugs is a topic of increasing interest. Recent evidence leads to the hypothesis that bevacizumab in combination with antibody‒drug conjugates (ADCs) could improve therapeutic effectiveness in OC using a complementary mechanism of action. Anti‐VEGF drugs temporarily normalise tumour vasculature, lowering interstitial pressure and facilitating ADCs intratumoural delivery, potentially increasing payload penetration and target engagement^32^ (Figure 2a). Several ongoing trials are investigating the role of combination with bevacizumab both in heavily treated recurrent OC, mainly with mirvetuximab soravtansine^33^ and luveltamab tazevibulin,^34^ and in the frontline maintenance setting, in particular with mirvetuximab soravtansine^29^ and trastuzumab deruxtecan.^35^

In addition, also the combination of bevacizumab with ICIs in OC is supported by a strong biological evidence: angiogenesis contributes to an immunosuppressive microenvironment, consequently VEGF blockade promotes vascular normalisation, enhances T‐cell infiltration and amplifies ICI activity^36^ (Figure 2b). The recent phase III trial KEYNOTE‐B96 showed improved PFS and OS with pembrolizumab plus chemotherapy ± bevacizumab in platinum‐resistant disease^37^ and encourage further exploration of these combination strategies. Even in this setting, the identification of biomarkers could ensure more accurate patient selection.

### 4.2 Biomarkers of bevacizumab response in human cancers

In the last 20 years, bevacizumab has been approved by FDA and EMA for the treatment of metastatic colorectal cancer (mCRC), non‐small cell lung cancer (NSCLC), renal cell cancer, breast cancer (BC) and OC. Many studies have tried to identify predictive biomarkers of response, but to date no one has been validated. Since the addition of bevacizumab to standard therapy has shown benefit, general population is still being treated without pre‐selection, but a predictive biomarker would avoid unnecessary costs and toxicities of treating patients who do not show upfront response.

The biomarkers investigated in the context of bevacizumab therapy have been primarily selected based on their biological involvement in VEGF‐driven angiogenesis, vascular stabilisation, hypoxia signalling and modulation of the tumour immune microenvironment. These molecules are hypothesised to influence sensitivity or resistance to VEGF blockade by reflecting either the dependency of the tumour on angiogenic pathways or the activation of compensatory mechanisms that may limit the clinical efficacy of bevacizumab.

Levels of VEGF‐A, the main target of bevacizumab, have been one of the first investigated biomarkers. Increased concentration of VEGF‐A in patient serum was generally correlated with poor prognosis in hepatocellular carcinoma,^38^ OC,^39^ BC,^40^ colorectal cancer (CRC)^41^ and gastric cancer (GC).^42^ However, no study has been able to demonstrate a predictive value for bevacizumab therapy response or to validate this finding in large cohorts in prospective trials,^43^, ^44^, ^45^, ^46^, ^47^ concluding that VEGF‐A levels can be useful as a prognostic rather than a predictive biomarker.

VEGF‐A alternative splicing generates several isoforms with different biological roles in angiogenesis; some studies have suggested that the analysis of specific isoforms or the ratio among them and total VEGF‐A levels could identify more specific biomarkers of bevacizumab efficacy in mCRC^48^, ^49^ and NSCLC.^50^ Moreover, VEGF gene polymorphisms have been investigated in CRC but no predictive impact was demonstrated.^51^, ^52^

Other angiogenic factors were also investigated, but no clear biomarker was identified. BC patients with low tumour levels of the angiogenesis regulators delta‐like canonical notch ligand 4 (Dll4), VEGF‐C and neuropilin‐1 (NRP‐1) may benefit from bevacizumab addition to chemotherapy,^53^ while tumour NRP‐1 levels were found to be good biomarker candidates in advanced GC.^45^ High tumour expression of the sprouting enhancer APLN^54^ and high plasma levels of placental growth factor (PlGF) and VEGF‐D^55^ were predictive of failure to respond to bevacizumab treatment in mCRC. A signature based on plasma levels of angiopoietin like 4 (ANGPTL4), hepatocyte growth factor (HGF) and VEGF‐A_121_ isoform could predict benefit from bevacizumab addition in mCRC.^56^ Another study identified a plasma signature consisting of high levels of epidermal growth factor and macrophage‐derived chemokine and low levels of IL‐10, IL‐6 and IL‐8 as associated with better response to bevacizumab in mCRC.^57^ On the other hand, angiogenic plasma biomarkers such as bFGF, E‐selectin, ICAM‐1, PlGF, VEGFR‐1 and VEGFR‐2 were not predictive of bevacizumab response in NSCLC.^46^

Other factors from the TME were investigated, but the studies were only able to demonstrate associations and not biomarkers with predictive value.

A signature with high expression of the tumor growth factor‐β (TGF‐β) superfamily member activin A, IL1β, and the urokinase plasminogen activator surface receptor correlated with response to bevacizumab treatment as monotherapy in metastatic melanoma.^58^

hERG1 potassium channels regulate the secretion of VEGF‐A and the process of neoangiogenesis; the presence of hERG1 in the tumour, together with the presence of the active form of HIF‐2α, was associated with lower risk of progression when bevacizumab was added to chemotherapy.^59^

VEGF stimulates the production of nitric oxide (NO). Monitoring of NO serum levels in 15 NSCLC patients before and during bevacizumab treatment demonstrated that reduced NO under bevacizumab was associated with a longer patients’ PFS, suggesting that NO can represent a dynamic biomarker of the response to bevacizumab. Of course larger series of patients and confirmatory studies are needed.^60^

Taken together, these reports indicate that there is no consensus on the role of angiogenic‐ and microenvironment‐related molecules in predicting the response to bevacizumab. The major limitations for most of the studies consist in the small sample size and lack of independent validation in subsequent work.

## 5 BIOMARKERS OF BEVACIZUMAB RESPONSE IN OVARIAN CANCER

**FIGURE 3:**
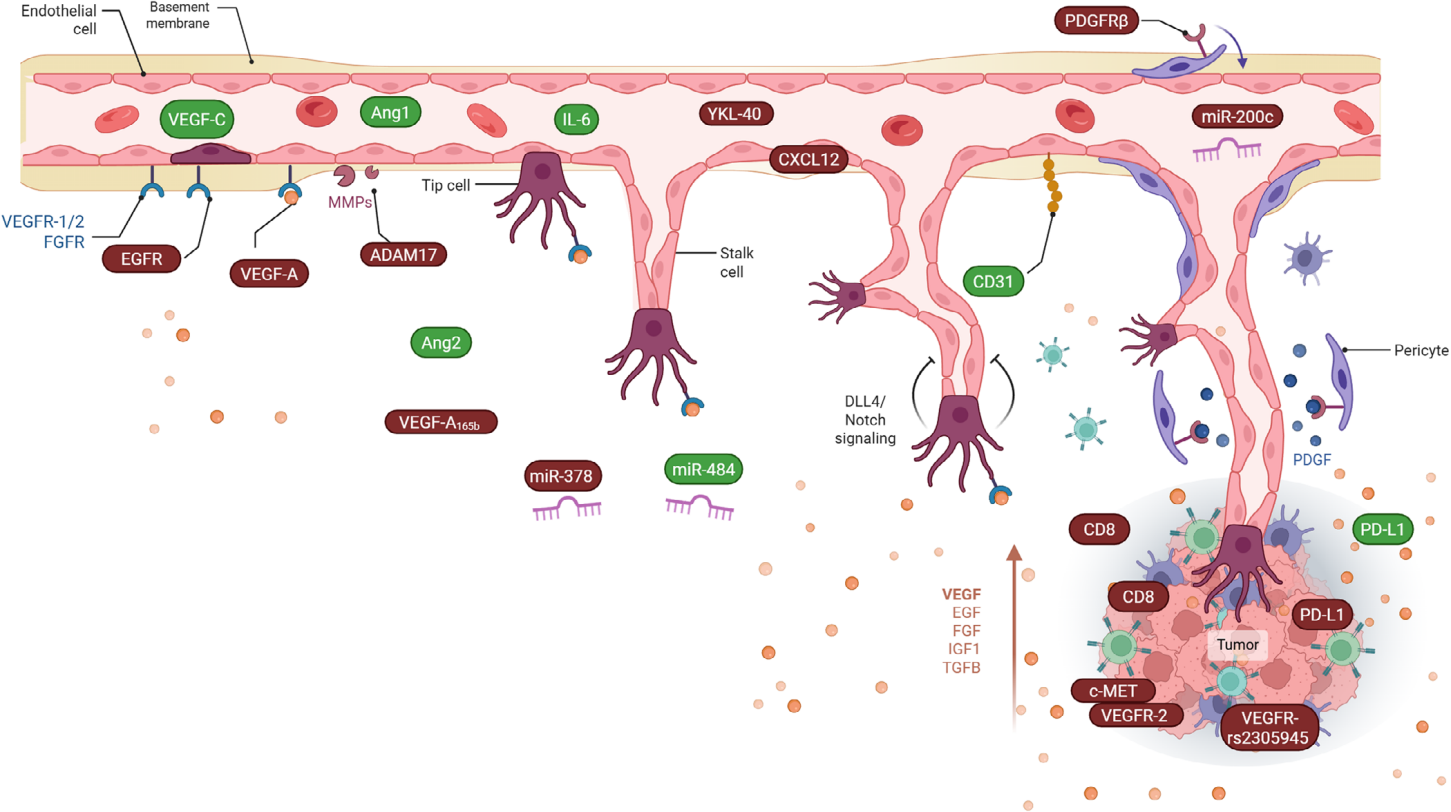
Schematic overview of the ovarian cancer tumour microenvironment and biomarkers of bevacizumab response. Scheme representing the studied biomarkers (in circles) in the context of tumour angiogenesis in ovarian cancer. Green circles and red circles indicate biomarkers associated with good or poor response to bevacizumab, respectively.

Great efforts by different research group have been dedicated to find predictive biomarkers of response in OC also using sample from large clinical trials (Table 3 and Figure 3). The target molecule was selected based on its biological relevance in OC angiogenesis, its mechanistic role in tumour growth and immune modulation, and the feasibility of its evaluation through translational and clinically applicable approaches. Below we summarise these results discussing the evidences on circulating, tissue and signature biomarkers and dedicate particular evidence to the work of the MITO group who recently designed and conducted the phase IV MITO16A/MaNGO‐OV2A clinical trial that aimed to specifically identify and/or validate prognostic biomarkers of bevacizumab‐treated OC patients.^61^

**TABLE 3:** Biomarkers evaluation in ovarian cancer.

	Biomarker	Enrolled patients	Technique	Notes
Circulating	FLT4, AGP, mesothelin, CA‐125^54^	217	LC‐MS/MS, ELISA	Gained a predictive value when combined with CA‐125 in a biomarker index; improved PFS in biomarker‐positive patients. ICON7 trial
	Ang1, Tie2^55^	91	Multiplex ELISA	High Ang‐1/low Tie2 levels associated with improved PFS benefit from bevacizumab. ICON7 trial
	Tie2^56^	92	Multiplex ELISA	Tie2 in combination with CA‐125 can identify progressive disease in bev‐treated patients. ICON7 trial
	VEGF‐C^59^	101	ELISA	High VEGF‐C levels predicted better probability of response to bevacizumab
	IL‐6^60^	751	Multiplex immunoassay	Patients with high levels of IL‐6 may benefit from treatment with bev
	YKL‐40^64^	140	ELISA	Low plasma YKL‐40 associated with better outcome in bev‐treated patients. GOG‐0218 trial
	NRP‐1^65^	980	IHC	No predictive value
	miR‐200c^68^	207	qPCR	Low miR‐200c levels predicts benefit from bev treatment. ICON7 trial
Tissue	VEGF‐A_165b_^70^	413	IHC	Low VEGF‐A165b associated with better outcome in bev‐treated patients. AGO‐OVAR 11
	c‐MET/VEGFR‐2 co‐localisation^71^	178	Immunofluorescence	High c‐MET/VEGFR‐2 co‐localisation correlated with worse PFS and OS in bev‐treated patients. ICON7 trial
	VEGFR‐2 rs2305945^71^	449	Genotyping	VEGFR‐2 rs2305945 correlated with worse PFS and OS in bev‐treated patients. ICON7 trial
	tVEGF‐A and CD31^65^	980	IHC	High MVD and high tVEGF‐A may predict benefit from bev treatment. GOG‐0218 trial
	Ang‐2^72^	205	Western blot, IHC, ELISA	High expression correlates with significant benefit from bev treatment
	PDGFR‐beta and VEGFR‐2^74^	391	Tissue microarray, IHC	No predictive value
	miR‐378^75^	421	In vitro experiments correlated with data from TCGA	Low miR‐378 expression correlated with longer PFS
Molecular signatures	Gene expression profiles^70^	359	Whole genome microarray analysis	Proliferative and mesenchymal molecular subtypes have a better response to anti‐angiogenic therapy. AGO‐OVAR11 trial
	FGFR1, FGFR4, FGF19^78^	380	NanoString	Presence of a signature (low FGFR1, low FGFR4, high FGF19) predicts higher bev efficacy. AGO‐OVAR11 trial
	EGFR, HER2 mutations^79^	62	NGS	Patients with EGFR or HER2 alterations did not respond to bev treatment
MITO16A	MVD, α‐SMA, VEGFA, VEGFR‐2, HIF‐1α^80^	336	IHC	No predictive value
	MicroRNA signature (miR‐484)^80^	336	miRNA expression	High miR‐484 expression associated with longer PFS and OS; combined miR‐484/VEGFB may identify patients benefiting from bevacizumab
	ADAM17^83^	309	IHC	Low ADAM17 score associated with better survival for bev‐treated patients
	EGFR membrane staining^84^	310	IHC	Strong membrane EGFR associated with worst outcome in bev‐treated patients
	TP53^85^	197	NGS sequencing and IHC	Unclassified TP53 missense mutations associated with better OS in bev‐treated patients
	HRD evaluation^86^, ^87^, ^88^	100	NGS	Concordance with commercial HRD testing
	miR‐506^88^	72	Microarray profiling	Can be coupled with HRD testing to define population with better prognosis
	CXCL12, CXCR4, CXCR7^92^	308	IHC	High CXCL12 associated with shorted PFS and OS
	PD‐L1^93^	292	Multiplex immunofluorescence	Absence of PD‐L1 in the tumour and presence of PD‐L1 in the stroma predict better outcome in bev‐treated patients
	CD8, CD68	292 171	Multiplex immunofluorescence Gene expression profiling	Immune infiltration is associated with shorter PFS in bev‐treated patients
	Polygenic toxicity risk^94^	171	Machine learning	Identified SNVs important for toxicity prediction in bev‐treated patients

Abbreviations: ADAM 17, a disintegrin and metalloproteinase 17; AGP, arabinogalactan‐proteins; Ang1, angiopoietin‐1; Ang2, angiopoietin‐2; bev: bevacizumab; Ca 125, cancer antigen 125; CD, cluster of differentiation; CXCL, C‒X‒C motif chemokine ligand; CXCR, C‒X‒C motif chemokine receptor; EGFR, epidermal growth factor receptor; ELISA, enzyme linked immunosorbent assay; FGF, fibroblast growth factor; FGFR, fibroblast growth factor receptor; FLT4, Fms‐related tyrosine kinase 4; HER2, human epidermal growth factor; HIF‐1, hypoxia‐inducible factor‐1; HRD, homologous recombination deficiency; IHC, immunohistochemistry; IL‐6, interleukin‐6; LC‒MS, liquid chromatography‒mass spectrometry; MIR, microRNA; MVD, microvessel density; NGS, next generation sequencing; NRP‐1, neuropilin‐1; OS, overall survival; PDGFR, platelet‐derived growth factor receptors; PD‐L1, programmed death‐ligand 1; PFS, progression‐free survival; qPCR, quantitative polymerase chain reaction; SMA, smooth muscle active; SNV, single nucleotide variant; TCGA, The Cancer Genome Atlas Program; Tie2, angiopoietin‐1 receptor; TP53, tumour protein 53; VEGF, vascular endothelial growth factor; YKL‐40, chitinase‐3‐like protein.

### 5.1 Circulating biomarkers (plasma/serum, cfDNA, miRNAs)

Mass spectrometry and immunoassays analyses on serum samples from 205 patients enrolled in the ICON7 trial allowed to identify three candidate biomarkers (the tyrosine kinase receptor FLT4 [VEGFR‐3], α1‐acid glycoprotein [AGP] and mesothelin), which were differentially expressed between responders and non‐responders. These biomarkers were not individually predictive of bevacizumab response, but gained a predictive value when combined with CA‐125 in a biomarker index.^62^ However, further studies are required to validate the usefulness of this index.

The levels of 15 angiogenesis‐associated proteins (Ang2, FGFb, HGF, PDGFbb, VEGFA, VEGFC, GCSF, IL8, KGF, PlGF, VEGFR1, VEGFR2, Ang1, Tie2 and VEGFD) were analysed in plasma samples from the same patients included in the ICON7 using multiplex enzyme linked immunosorbent assays (ELISAs). The Ang1/Tie2 axis is a key pathway that regulates vascular stability. A combination of Ang1 and Tie2 expression was identified as predictor of bevacizumab response, but with minimal benefit advantage.^63^ Subsequent confirmatory analyses on 650 serum samples from 92 patients showed that Tie2 increase in combination with CA‐125 was shown to be a more effective biomarker.^64^ Tie2 was also confirmed to be a valuable response biomarker for VEGF inhibitors in mCRC^65^ and is currently being tested in a prospective non‐interventional biomarker study that will hopefully provide more definitive insights on the value of the Tie2 test to predict the response to bevacizumab.^66^ This is consistent with the fact that VEGF inhibition often leads to a deregulation of the Tie2 axis.

A single report that collected blood samples from primary epithelial ovaria cancer (EOC) patients at surgery showed that increased serum levels of VEGF‐C predicted higher response to bevacizumab,^67^ but further studies are needed to confirm this hypothesis.

Analysis of plasma levels of IL‐6, Ang‐2, OPN, SDF‐1 (CXCL12), VEGF‐D, IL‐6R and GP130, all vascular modulators, on samples from patients enrolled in GOG‐0218 trial,^18^ revealed that high IL‐6 levels may define patients most likely to benefit from the addition of bevacizumab to standard chemotherapy.^68^ This is in agreement with an important role of IL‐6 in defining the inflammatory TME in OC.^69^

The proangiogenic secreted glycoprotein YKL‐40 is an emerging prognostic biomarker for different types of cancer and its plasma levels are increased in OC patients compared to healthy subjects, and even more increased in metastatic settings.^70^, ^71^ Low plasma levels of YKL‐40 predicted improved PFS and OS outcome in patients with advanced OC treated with bevacizumab, although these results need validation in bigger cohorts.^72^

High NRP‐1 expression was proposed to predict poor prognosis in OC patients, consistent with the role of NRP‐1 as an enhancer of VEGF signalling.^73^ As a tissue biomarker, it failed to predict bevacizumab response in a retrospective analysis of the GOG‐0218 clinical trial^74^; however, research is ongoing to determine if soluble NRP‐1 could represent a circulating biomarker to predict bevacizumab response.^75^

A tumour suppressor miRNA, miR‐200c, has been linked with EMT and resistance to chemotherapy.^76^ Low level of miR‐200c in plasma can predict patients who can benefit from bevacizumab addition, but validation is necessary.^77^ Predictive and prognostic biomarkers are under investigation also in tumour‐derived cfDNA from plasma and ascites of OC patients.^78^ Ascites in particular has been found to contain abundant cfDNA and could represent a source of potential biomarkers.

### 5.2 Tissue biomarkers (immunohistochemistry and spatial analyses)

Tissue samples collected in the ICON7 trial identified VEGF‐A_165b_ as an ‘antiangiogenic’ isoform that could contribute to poor response to bevacizumab treatment. Patients with low levels of VEGF‐A_165b_, detectable by IHC, showed improved OS when bevacizumab was added to standard platinum/paclitaxel‐based chemotherapy.^79^

Using a tissue microarray prepared using a subset of samples from patients enrolled in ICON7 trial, it has been shown that high c‐MET/VEGFR‐2 co‐localisation on tumour tissue was shown to be associated with poorer survival outcomes in bevacizumab‐treated patients; moreover, from the same trial, patients bearing the specific VEGFR‐2 rs2305945 G/G variant had shorter PFS when treated with bevacizumab.^80^ However, this result needs to be validated in larger cohorts.

Consistently with the bevacizumab mechanism of action, tumoural VEGF‐A expression and microvessel density (MVD), evaluated by IHC using the expression of the endothelia marker CD31, showed potential predictive value in retrospective tumour biomarker analyses of the GOG‐0218 trial.^74^ These data merit future confirmation from prospective randomised trial.

Different other biomarkers were tested in samples collected from patients not included in randomised trials. Analyses of Ang‐2 expression on tumour tissue using Western blot and IHC showed that high expression correlates with significant benefit from bevacizumab therapy.^81^

High levels of PDGFR‐beta and VEGFR‐2 have been correlated with platinum resistance and poor prognosis.^82^ The same group has recently tried to associate stromal levels of these factors, analysed by tissue microarray IHC, with predictive value for bevacizumab treatment; consistent with the previous results, high expression of PDGFR‐beta was associated with shorter OS, but this was not specific to the group of bevacizumab‐treated patients. Bevacizumab treatment led to better prognosis in all patients, regardless of PDGFR‐beta levels.^83^ VEGFR‐2 protein levels were almost absent in tumour samples, and this correlated with improved OS, but again not only in bevacizumab‐treated patients, suggesting that optimal bevacizumab response does not rely on VEGFR‐2 expression.^83^

Vascular‐associated microRNAs, also known as angiomiRs, have been shown to regulate angiogenesis and metastasis. miR‐378 has been shown to promote angiogenesis in various cancers. In vitro experiments correlated with data from The Cancer Genome Atlas Program (TCGA) showed that low miR‐378 expression was predictive of response to combination bevacizumab and chemotherapy.^84^ miR‐6086 has been shown to inhibit angiogenesis in OC by impacting the OC2/VEGFA/EGFL6 axis and could be used as a biomarker.^85^ In addition to miRNAs, regulation of angiogenesis‐related processes by other epigenetic mechanisms has been gaining attention. Inhibition of the epigenetic modifiers BRD2/3/4 belonging to the BET family of proteins has been shown to regulate the response to antiangiogenic therapy.^86^ Further investigation could be useful to identify biomarkers of response and resistance to bevacizumab. Indeed, a study reports that BET inhibitors demonstrated efficacy in overcoming resistance to radiotherapy in OC, suggesting the role of the BET‐regulated gene *GNL3* as a biomarker for selecting personalised therapy.^87^

### 5.3 Molecular signatures/genomic and transcriptomic classifiers

Molecular signatures derived from gene expression profile (GEP) studies divided HGSOC in four different subtypes association with prognostic significance.^88^, ^89^ Application of these signatures to a subset of patients from the ICON7 trial found a correlation between molecular subtype^88^ and response to anti‐angiogenic therapy, showing that the mesenchymal and proliferative subtype, those with poorest survival, have a better response to anti‐angiogenic therapy.^79^

A study tried to correlate patient outcome with the expression of the fibroblast growth factor receptors and their ligands (FGFRs/FGFs), a family of proteins involved in angiogenic signalling. A signature based on the expressions of FGFR1, FGFR4 and FGF19 was able to predict favourable prognosis for bevacizumab‐treated patients.^90^

Finally, genetic alterations of EGFR or HER2 were found to be associated with poor response to bevacizumab plus chemotherapy.^91^

Overall, our literature search did not identify validated biomarkers of response to bevacizumab, which could be ready for clinical application. Serum biomarkers would represent the most promising, allowing a minimally invasive detection from patients’ plasma. However, all the studies we examined suffer from the lack of external validation, either in bigger cohorts or in biomarker‐driven prospective trials. Moreover, lack of consistency could be also due to poor reproducibility: the assays used in single laboratories are not standardised in terms of cut‐offs or statistical analyses.

## 6 PROGNOSTIC BIOMARKERS OF RESPONSE TO BEVACIZUMAB IN OVARIAN CANCER: RESULTS FROM MITO16A/MANGO OV‐2 TRIAL

**FIGURE 4:**
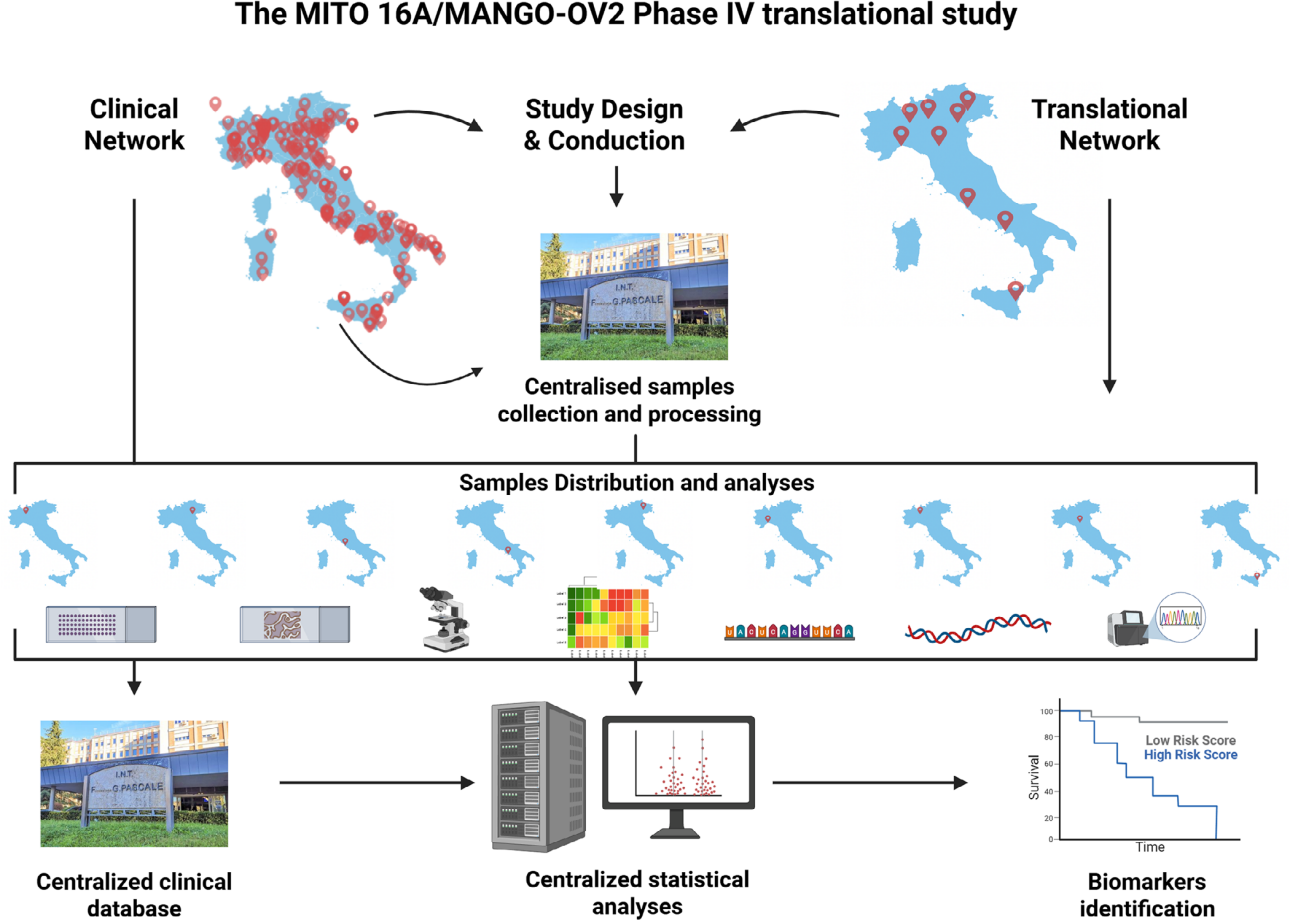
Schematic workflow of the MITO16A/Mango OV2 trial. The MITO clinical and translational network jointly designed the translational endpoints of the trial looking at the available information and personal unpublished data. After the completion of patients’ enrollment, a review of the anticipated biomarkers has been done. Samples collection and processing (tissue micro‐array and whole slide preparation, nucleic acid extraction) have been centralised to ensure quality control of used material. The clinical database was also centralised. Processed tissues were distributed to the peripheral centre that performed locally the analyses of selected biomarkers. All clinical and molecular data were then processed for prognostic and statistical analyses by a centralised group of statisticians in blind to ensure the reliability of the results.

To establish a standardised system to identify biomarkers of bevacizumab response, the MITO group in Italy has specifically conceived the MITO16A/MaNGO OV‐2 trial. The multicentre, phase IV, single‐arm MITO16A/MaNGO OV‐2 trial was designed to explore molecular and clinical biological factors allowing to identify OC patients who could benefit from the addition of bevacizumab to standard chemotherapy in first line.^61^ The biomarker‐driven trial enrolled 398 patients who all received bevacizumab with first‐line chemotherapy. One limitation of the study was the absence of a chemotherapy‐only control arm, which allowed for the identification of prognostic biomarkers within a bevacizumab‐treated cohort, but prevented the evaluation of predictive biomarkers. Tissue samples collection was centralised at the INT G. Pascale of Naples that supervised the quality controls and performed tissue processing to build tissue micro‐array (TMA), to extract nucleic acid and provide final tissue slides to investigators as necessary (Figure 4). Biomarkers analyses included the evaluation of protein expression on TMA or tissue slides, depending on the biomarker in study, the evaluation of gene and microRNA expression and the analyses of DNA somatic alterations including HRD. To ensure the reliability of the data, the MITO‐group researchers agreed to perform the wet analyses locally and have a central independent statistical evolution of the results that followed rigorous statistic pipelines and avoided data torturing (Figure 4).

The first attempt was to confirm a possible prognostic role for angiogenesis‐related molecules such as MVD evaluated using CD31 expression, α‐SMA, VEGFA, VEGFR‐2 and HIF‐1α that were assessed on tissue slides. However, once correlated with clinical variables and patients’ outcome, these markers lost significance.^92^ Due to the design of the study in which all patients received bevacizumab treatment we could not define the predictive value of tissue VEGFA and MVD proposed by Bais et al.^74^ Analysis of an angiogenesis‐related microRNA signature, and in particular miR‐484, a regulator of VEGFB and VEGFR‐2 pathways,^93^ did not show significant correlation with MVD, although high levels of miR‐484 were observed in VEGF‐B‐negative samples, confirming previous observations.^93^ As a possible hint for future investigation it has been observed that the association of high miR‐484 levels and high VEGF‐B expression was associated with longer PFS.^92^ Regarding the expression of VEGFR‐2, the work confirmed that this receptor is generally not expressed by tumour cells, as observed by others,^83^ and that therefore the use of TMAs is not a valuable tool to evaluate its expression in OC.^92^

A disintegrin and metalloprotease 17 (ADAM17) is involved in OC progression and cisplatin resistance.^94^ A low ADAM17 score in the examined patients was associated with better survival; however, the prognostic value lost significance after statistical correction for multiple testing and could be prognostic only in the subgroup of patients without residual disease at baseline.^95^

ADAM17 mediates activation of EGFR signalling through proteolytic cleavage. EGFR overexpression in OC has been associated with poor prognosis. Combination of GEP and IHC studies demonstrated that strong and homogeneous EGFR membrane staining by IHC were associated with the worst outcome after bevacizumab treatment. This group of patients was correlated with activation of EGFR pathways and lack of angiogenic‐related molecular features.^96^

Next‐generation sequencing (NGS) sequencing and IHC studies were used to evaluate the possible prognostic role of *TP53*, the gene most frequently mutated in OC. *TP53* mutational status was assessed by NGS and correlated with p53 expression evaluated by IHC. In the MITO16a case material, the presence of *TP53* mutations (in particular unclassified missense mutations) was associated with increased OS benefit in patients receiving bevacizumab in addition to standard chemotherapy independently of clinicopathological characteristics. On the other side, TP53 status evaluated by IHC did not have prognostic significance in the same case group.^97^ These results highlight the possible different significance of diverse TP53 mutation and warrant future confirmatory investigations.

NGS approaches were also used to evaluate the prognostic significance of HRD in a subset of patients enrolled in the MITO16A trial using commercial and academic approaches. All collected data demonstrated that evaluation of HRD using NGS approaches had prognostic significance also in patients treated with bevacizumab in first line settings.^98^, ^99^, ^100^ On the other side, the evaluation of HRD using a functional approach based on the evaluation of RAD51 expression by immunofluorescence^101^ did not provide conclusive prognostic indication in the studied population. However, the combined evaluation of functional and genomic HRD coupled with the expression of miR‐506 may be used to further refine HRD status and identify a group of patients with particular good prognosis.^100^

A growing body of evidence supports the idea that bevacizumab can modulate the complex tumour immune microenvironment in OC. Therefore, immune‐related molecules could be relevant biomarkers of the response to bevacizumab treatment.^102^

The CXCR4‒CXCL12 chemokine axis plays a significant role in OC by promoting cell proliferation, migration, invasion and metastasis. It also contributes to reshape the TME, mainly by altering the immune responses.^103^ Epithelial and stromal expression of CXCL12 and its receptors CXCR4 and CXCR7 was evaluated by IHC on 308 EOC samples included in the MITO16A TMA. Among these proteins, only high epithelial CXCL12 was negatively associated with PFS and OS, although the statistical significance was lost after adjusting for overfitting.^104^

Full sections from 292 patients were analysed by a multiplex immunofluorescence (MIF) approach applied to the MITO16A case material. This allowed the evaluation of the levels and the spatial localisation of programmed death‐ligand 1 (PD‐L1), tumour cells (cytokeratins), T cells (CD8) and monocyte/macrophage populations on a single tissue slide. The absence of PD‐L1‐positive cells in the tumour and the presence of PD‐L1‐positive cells in the stroma were associated with a better prognosis for patients treated with chemotherapy plus bevacizumab, although the associations lost significance after statistical correction.^105^

The prognostic value of the immune infiltrate was further examined coupling spatial MIF analysis of CD8^+^ and CD68^+^ cells and molecular stratification of patients through gene expression profiling. The results suggest that immune‐infiltrated tumours responded less to bevacizumab treatment, although this finding needs to be confirmed with prospective trials.^106^ If validated in future prospective investigations, this observation would hold substantial translational significance. Tumour immune infiltration, particularly by CD8^+^ T lymphocytes, is a well‐established positive prognostic marker in OC patients treated with bevacizumab‐free regimens. Therefore, systematic assessment of the immune infiltrate may enable oncologists to stratify patients according to the likelihood of deriving clinical benefit from the addition of bevacizumab, while also identifying those for whom such treatment may be ineffective or even detrimental with respect to PFS.

These works demonstrated the feasibility and utility of using MIF in large clinical trials in OC and support the possibility that spatial distribution of single biomarkers could have different prognostic value in cancer, adding complexities to the field. For these reasons we believe that machine learning (ML) and artificial intelligence (AI) approaches might help in the evaluation and integration of multiple variables providing a clearer and more reproducible picture of biomarkers prognostic/predictive roles in human cancer. In a recent multicentre study, deep‐learning models applied to digital histopathology slides accurately estimated a molecular signature associated with sensitivity to atezolizumab–bevacizumab, identifying patients with significantly improved PFS. This fast and scalable approach, potentially extensible to other solid tumours, including OC, suggests that AI‐driven models may support the development of non‐invasive predictive biomarkers for anti‐VEGF treatments such as bevacizumab.^107^ We started to apply a ML approach on germline DNA variants associated with drug‐induced toxicities in 171 patients with OC enrolled in the MITO16A trial and demonstrated that ML identified a polygenic toxicity risk score able to better categorise the patients into high‐ and low risk of drug toxicities.^108^ Several other studies have already shown concrete applications of ML/AI in biomarker prediction and clinical decision support, such as radiomic models capable of predicting BRCA/HRD and neural networks that classify microenvironmental immune patterns with prognostic value. This evidence further supports the utility of advanced computational approaches in integrating heterogeneous variables and improving the accuracy of patient stratification.^109^

In conclusion, despite the limitations of the study, the analyses of biomarkers from the MITO16A/MaNGO OV‐2 trial allowed to identify key factors affected by bevacizumab treatment. The collected data can represent a valuable resource for the scientific community, supporting the work of other researchers to focus on specific pathways and identify predictive biomarkers of bevacizumab response in OC patients. In addition, the robust statistical pipeline exploited by the MITO group represents an example of rigorous statistical analysis of clinical biomarkers.

## 7 CONCLUSION AND FUTURE PERSPECTIVE

The most recent literature reviewed shows two main limits for biomarkers validation: rarely the same biomarker was tested using the same approach in different studies by independent researchers and only few studies were based on analyses of samples from patients included in clinical trials with translational endpoints. The adding value to have patients with similar clinical characteristics and homogenously treated in a controlled clinical trial should not be underestimated. The MITO group tried to deal with these two limitations by designing and conducting a prospective phase IV trial whose principal aim was to validate biomarkers emerged from smaller cohorts of patients or based on biological rationales from preclinical studies. Many of the initially promising biomarkers did not retain statistical significance once the analyses were adjusted for multiple testing using appropriate shrinkage methods. A rigorous statistical correction is therefore essential to ensure that only truly robust biomarkers progress to further validation, ultimately preserving time, biological samples and financial resources and to avoid overestimating the relevance of individual signals and generate false‐positive findings.

The identification of robust prognostic and predictive biomarkers could enable a more targeted and effective use of bevacizumab, enhancing the therapeutic potential of its latest combinations with innovative agents, including ADCs, ICIs and PARPi. Biomarkers such as HRD, for example, may help guide treatment selection and sequencing, optimising the use of targeted therapies and maximising the clinical benefit of these emerging combinatorial strategies.

In conclusion, we want to point out that the use of novel technologies such as spatial proteomics and transcriptomics along with the more and more widespread use of nucleic sequences produce compelling and potentially relevant data and could be of full clinical utility when integrated with novel approaches of ML and AI. In this context, spatial transcriptomics and multiplexed proteomics could enable the spatially resolved identification of angiogenic, stromal and immune signatures, which, when integrated into AI‐driven models, may significantly improve biomarker discovery and bevacizumab response prediction in future trials.^110^ This could allow also the integration of multiple biomarkers to generate ‘complex signatures’. However, these new technologies are still at research grade, and accurate standardisation would be required for the application of these techniques in diagnostic settings. Based on these considerations, we believe that the future of cancer research requires the complete integration of multidisciplinary expertise that is particularly possible in the frame of large cooperative clinical and translational research groups.

## AUTHOR CONTRIBUTIONS

The authors have nothing to report.

## CONFLICT OF INTEREST STATEMENT

S.P. received honoraria from Roche, AZ, MSD, GSK and research funding from Roche, AZ, MSD, GSK and Pfizer. M.R.L., E.P., G.B., C.D.A., D.L. and F.B.V. declare they have no conflicts of interest.
